# Mycofabrication of sustainable mycelium-based leather using *Talaromyces* sp. and irradiated eggplant peel waste

**DOI:** 10.1186/s13568-025-01935-0

**Published:** 2025-08-22

**Authors:** Reham M. M. Abdelkader, Ola M. Gomaa

**Affiliations:** Radiation Microbiology Department, National Center for Radiation Research and Technology (NCRRT), Egyptian Atomic Energy Authority (EAEA), Cairo, Egypt

**Keywords:** *Talaromyces* sp., Fungal mycelium, Alternative leather, Food waste valorization, Tensile strength

## Abstract

Agro-industry and leather manufacturing are considered two of the most polluting industries worldwide due to the huge amount of waste they produce that contributes to pollution. To address the challenges of food waste while contributing to sustainable leather production, this study explores the feasibility of developing an alternative, ecofriendly leather material. *Talaormyces* sp. was isolated from eggplant peel waste and was identified using phylogenetic ITS region (Genbank accession number: PQ007745); the closest relatedness was with *Talaromyces atroroseus.* Eggplant peel waste was exposed to electron beam irradiation at 5, 10, 15, 20, and 25 kGy. The results showed that exposure of eggplant peel waste to electron beam irradiation affected its rigidity, color, and tensile strength, where exposure to 15 kGy resulted in flexible material that showed tensile strength of 8 MPa, whereas non-irradiated sheets showed tensile strengths of 5 MPa. Modification and optimization of the cultivation media were performed using Full Factorial Design of Experiment. Sucrose (20, 40 g/L), calcium chloride (0.5, 1 M), and glycerol (10, 20%) were tested as 3 factors, two levels. The results showed that increasing the concentration of sucrose in the cultivation media from 20 to 40 g/L increased the mycelial network and induced exopolysaccharides, which act as the bio-binder in the mycelial-leather forming process. A post-treatment study was performed to ensure that there are no spores in the myco-product. These findings demonstrate that both fungal mycelium and eggplant waste-derived fungal sheets have great potential as ecofriendly materials with leather-like properties.

**Supplementary Information:**

The online version contains supplementary material available at 10.1186/s13568-025-01935-0.

## Introduction

Food waste makes up the largest share of waste disposed in landfills due to its high moisture content and susceptibility to biodegradation, both presenting significant challenges, and is distinguished by its microbial instability and high moisture content, which present challenges in its management, regardless of its source (Nayak and Bhushan 2019). Moreover, food waste decomposition in landfills emits gases which contribute to the greenhouse effect. According to the Food and Agriculture Organization (FAO), it is estimated that a third of the food produced for human consumption, 1.3 billion tons each year, is wasted or lost all over the food chain. Losing food implies that there is unnecessary pressure on land, energy, water, agricultural reserves, packaging, and transport, which reduces resources and contributes to the environmental pollution problem and greenhouse gases (GHGs) released, which consequently lead to more food decomposition (FAO 2019).

Eggplant, *Solanum melongena L*., belonging to the *Solanaceae* family, is one of the important crops that holds importance for cultivation since it produces high production yield (Gürbüz et al. 2018; Horincar et al. 2020; Agregan et al. 2021). It was estimated that the worldwide production quantity of eggplant was 58,646,098.21 tons in 2021 (FAOSTAT 2023). Despite the importance of these products, industries manufacturing such products are responsible for creating paramount waste of eggplant by-products, which are disposed of in landfills. The main byproducts of eggplant are peel and calyx. The peel is considered a rich source of anthocyanins (Mauro et al. 2020). Additionally, both peel and calyx of eggplant are great sources of dietary fibers, such as cellulose and pectin (Kazemi et al. 2019a; 2019b). Eggplant peels represent a significant portion of the vegetable, and their repeated disposal contributes to food waste (Lazar˘ et al. 2024).

On the other hand, the fashion industry is considered one of the most polluting industries worldwide, especially since consumption has increased in the past two decades (Woodside and Fine 2019; Shirvanimoghaddam et al. 2020). The global fashion market has increased from $1.5 trillion in 2020 to about $2.25 trillion in 2025 (Ikram et al. 2022; Papamichael et al. 2022). The fashion industry annually needs 79 billion cubic meters of water (about 20% of the world’s total water consumption), brings about 1.7 billion tons of CO_2_ (almost 10% of the world’s total CO_2_ emissions), and results in 92 million tons of textile waste (Sajn 2019). Leather products in particular are among the commonly traded products. The global leather goods market was estimated at 242.85 billion USD in 2022, with expectations to expand at a compound annual growth rate (CAGR) of 6.6% from 2023 to 2030 (Grand view research 2023) (http://www.grandviewresearch.com/). Leather manufacturing depends on animal farming that uses ploughable land, tons of animal feed, water, healthcare, and produces methane emissions, which make the leather industry an environmental threat (Roh 2020). Therefore, alternative and sustainable leather-like materials are highly demanded, especially with the rise in climate change awareness and vegan-based commodities (Majeed et al. 2023). Adopting sustainable practices would align myco-production with key sustainable development goals (SDGs). It reduces the use of harmful chemicals, thereby promoting good health (SDG 3: Good Health and Wellbeing), provides innovative material that supports industry and infrastructure (SDG 9: Industry, Innovation and Infrastructure), promotes sustainable manufacturing, and reduces waste generation (SDG 12: Responsible consumption and production). Additionally, it helps mitigate Greenhouse Gases (GHG) emissions. (SDG: Climate action). Other SDGs are also addressed, for example, it creates job opportunities and drives economic growth (SDG8: Decent work and economic growth). It also minimizes water consumption or producing polluted water (SDG 6: Clean water and sanitation) (https://sdgs.un.org/goals).

There are different leather substitutes derived from biobased materials that offer ecofriendly and novel techniques for fabrication (Majeed and Iftikhar 2024). One of the alternative leather substitutes is the one based on fungi as the substrate material. The successful fabrication of fungal based leather has led to commercial products that found their way to the market, such as Mylo™ which are mycelia grown on sawdust, ZAO™ which is collagen and genetically modified yeast, PinaTEX® which is made of mycelia grown on pineapple waste and MycoTEX® which depends on growing mycelia within specific molds to form the desired products. This has resulted in the creation of new start-up companies that focus on developing what is now known as vegan leather or sustainable leather alternatives. Based on their inherent properties of fungal mycelia and their biodegradability, they are easily composted, thus contributing to waste minimization and encouraging a bio-circular economy (Liu et al. 2024). Most of the commercialized alternative leather is based on white rot fungi, which comprise mainly filamentous fungal species of the Basidiomycota phylum, which belong to the fungal subkingdom Dikarya, also known as higher fungi (Amobonye et al. 2023). However, many species used in alternative leather, such as *Pleurotus ostreatus* (Oyster mushroom), *Pleurotus djamor* (pink mushroom), *Agaricus bisporus* (Button mushroom), and *Ganoderma lingzhi* (reishi) (Crawford et al. 2024) are edible and considered an affordable source of protein in many countries. This is considered non-feasible in many developing countries. From this standpoint, the present work aims to produce sustainable mycoleather using a local non-edible fungal isolate and irradiated eggplant peel waste as both a cheap biodegradable scaffold and source of nutrients. Moreover, post-treatment tests are conducted to ensure the safety of the mycoproduct. This research is the first to use eggplant peel waste to convert it to an alternative and sustainable material and the first to use gamma radiation at the pre- and post-fabrication stages.

## Material and methods

### Isolation and pure culture maintenance

Eggplant peel waste from vegetable vendors, Cairo, Egypt, was obtained and used for isolation of exopolysaccharide (EPS) producing fungi. The isolation was carried out by serial dilution and pour plate method using Sabouraud Dextrose Agar (SDA) as isolation medium. The plates were incubated for 7 days at 30 °C in the dark. Three isolates were obtained and were purified on SDA for three consecutive cycles. Pure cultures were maintained on SDA agar slants at 4 °C for further studies.

### Preparation of spore suspension and cultivation media for fungal selection

Spore suspension was prepared by growing fresh stock cultures on prepared Czapek’s yeast extract agar medium (CYA) Petri-dish at 28 °C ± 1 for 7 days. The spores were collected by adding 10 ml of NaCl (1% w/v) containing 5% Tween-80 (w/v) solution. Decimal dilution was prepared in sterile peptone physiological serum to obtain concentrations of 10^7^ spores/ml (Abdelkader et al. 2020). The prepared spore suspension was used to inoculate Yeast Extract Sucrose (YES) media, which contained the following in g/L: Sucrose 20, Yeast extract 4, MgSO_4_ 0.5, KH_2_PO_4_ 1, pH 6.2. About 1M CaCl_2_ was added to the media to induce stress, which resulted in EPS production (Gomaa et al. 2013, 2022). After incubation, cultures were filtered to remove fungal mycelia, and cold ethanol was added to the culture filtrate to precipitate exopolysaccharides (EPS) (Gomaa et al. 2022). One fungal isolate was chosen as the highest EPS producer and was used in the following experiments.

### Morphological identification

The promising fungal isolate was assessed for colony morphology on yeast extract sucrose agar (YES) medium. The colony characteristics and microscopic features, such as conidia, conidiophores, and size and shape of hyphae, were examined using a light microscope (Labomed, Labo America, Inc. USA) through slide cultures. The morphological features of the fungal isolate were also observed using scanning electron microscopy. The images were captured using a Zeiss evo15 SEM (Germany) located in the central laboratories at National Center for Radiation Research and Technology (NCRRT).

### Phylogenetic identification of fungus by ITS region

DNA extraction from samples was performed using QIAGEN DNeasy Plant Mini kit (Qiagen, Germany, GmbH) according to manufacturer’s instructions. PCR was performed using ITS 1 and ITS 4 with primer sequences TCCGTAGGTGAACCTGCGG and TCCTCCGCTTATTGATATGC, respectively (Salim et al. 2022). Amplification was performed as follows: denaturation at 94 °C for 5 min, amplification for 35 cycles at 94 °C for 45 s, annealing at 56 °C, extensions at 72 °C for 45 s, and final extension at 72 °C for 10 min. The visualized band-amplified PCR products were submitted to Solgent Co. Ltd (South Korea) for purification and sequencing. The sequence was compared to the NCBI nucleotide database (https://blast.ncbi.nlm.nih.gov/blast.cgi). Newick file was generated using the free online software www.phylogeny.fr. Molecular Evolutionary Genetics Analysis software (MEGA X) was used to construct the phylogenetic tree using the neighbour-joining method. The sequence of the fungal ITS was submitted to GenBank public database with accession number PQ007745. The strain was identified as *Talaromyces atroroseus* and was deposited in the Culture Collection Ain Shams University (CCASU), (10.12210/ccinfo.1186), Faculty of Pharmacy, Ain Shams University, PO Box 11566, Cairo, Egypt. Deposition number: CCASU-2025-F13.

### Electron beam accelerator irradiation of eggplant peel waste

The irradiation of samples was carried out at doses 0, 5 kGy (3 mA, 10.8 m/min), 10 kGy (5 mA, 8.86 m/min), 15 kGy (7 mA, 6.63 m/min), 20 kGy (10 mA, 22 m/min), and 25 kGy (12 mA, 23 m/min) in plastic bags using an Insulated Core Tank (ICT) electron beam accelerator (3 MeV, Energy 90 kw, Beam ampere Max up to 30 mA, Max conveyor speed 16 mm/min) at the National Center for Radiation Research and Technology, Cairo, Egypt.

### Mechanical tensile test

This test was conducted in the Main laboratory chemical warfare department according to the ASTM D638- 14 on 3 specimens for each sample to secure a 95% level of confidence. Each sample has 1 × 5 cm dimensions. To evaluate the tensile properties of the tested samples, a Tinius Olsen universal tensile testing machine model (H5KT) was used; the test was conducted at room temperature with a load cell 5 kN and crosshead rate 5 mm/min.

### Sensory analysis

Overall acceptability based on color, texture, and odor was performed by a trained panel of 5 members who participated in this study and were trained prior to the study. The tested samples (10 gm) were coded, placed in a sterilized Petri dish (90 mm), and presented to the panelists. The following attributes were investigated for each sample using a 4-point scale where 4 excellent, 3 good, 2 fair, and 1poor. The limit of acceptability was kept as 2.5, and the samples whose acceptability values were below 2.5 were rated unacceptable. The panel test was carried out under normal light conditions. The panelists were directed to estimate the odor of the samples by smelling the samples properly and determining the score as per the 4-point scale. Overall acceptability was presented as the mean value of the triplicate values of odor, color, and texture. The overall acceptability samples, which showed retention in creamy or white color, fresh-like texture, and odor, were rated higher than the samples which recorded a decrease in creamy or white color, mealy-like texture, and occurrence of off odor and excessive browning (Hussain et al. 2014).

### Total bacterial and fungal count

Total bacterial count (TBC) and total mold and yeast count (TMYC) were determined using pour plate technique by the serial dilution method cited by APHA (1992). A series of decimal dilutions of original samples was prepared with sterile saline solution. One ml of each appropriate dilution was mixed with 15 ml nutrient agar and Sabouraud agar media to determine TBC and TMYC. The samples were incubated at 37^o^ C for total bacterial count for 24 h, and at 30 °C for 5–7 days for total mold and yeast counts. Each value represents the mean of three samples and is expressed as logarithm colony-forming units per gram (log CFU/g).

### Full factorial design

The design of experiment (DOE) was performed using Minitab 20 software. Factors tested (3^2^) were a level three factors design, which were: Sucrose (20 and 40 g/L), CaCl_2_ (0.5 and 1 M), and glycerol (10 and 20%) as represented in S1. *Talaromyces* sp. spore suspension was prepared as mentioned above and used to inoculate the cultures as prepared in S1. The culture flasks were incubated at 30 °C for 7 days. Fungal mycelia were separated from culture filtrate. 2 vol 95% cold ethanol was added to the latter and left at 4 °C overnight. The precipitate was centrifuged at 4 °C and centrifuged at 6000 rpm for 20 min. The stickiness of the exopolysaccharides content was estimated by calculating the Protein/Carbohydrate ratio (P/C). Protein was assayed using the Bradford assay, and carbohydrates were assayed using a phenol sulfuric method as mentioned in a previous study (Maghrawy et al. 2024). Full Factorial Response represents stickiness of produced exopolysaccharides from each media.

### Optimized mycoleather preparation

Based on the previous experiments, eggplant peels (10 gm) were irradiated at a dose of 15 kGy as Irradiation was performed as mentioned above. Eggplant peel waste was placed in Petri dishes. About 25 ml YES media (8) was poured with 0.5 ml of spore suspension (10^7^), then incubated for 7 days at 30 °C for preparation of a new batch of mycoleather.

### Assessment of post-treatment on viable count of mycoleather and thermal analysis

Based on the full factorial design experiment and the tensile strength of irradiated eggplant peel, a new batch of mycoleather was prepared and was exposed to post-treatment which were as follows: (1) incubation at 40 °C, (2) exposure to electron beam radiation at dose 5 kGy (5 mA, 10.8 m/min) in Petri dishes using an ICT liner electron beam accelerator (3 MeV), Energy 90 kw, Beam ampere Max up to 30 mA, Max conveyor speed 16 mm/min, Max high voltage (3000 kv) at the National Center for Radiation Research and Technology, Cairo, Egypt, 3) exposure to UV (254 nm) at 20 cm distance for 60 min, and 4) immersion in 5% sodium hypochlorite. At the end of the treatments, viable count, sensory feel, and SEM were performed on the samples, as mentioned earlier. In addition to that, a Thermal Gravimetric Analysis (TGA) test was performed on the 4 samples using a Shimadzu (TGA-50). The samples underwent heating from room temperature to 600 °C at a heating rate of 10 °C/min under a nitrogen atmosphere with a flow rate of 20 ml/min.

### Statistical analysis

All experiments were conducted with three independent replicates, and results are presented as mean ± SD (n = 3) to ensure robust data collection. Data were analyzed using the SAS software package, version 9 (SAS, 2002). Statistical significance between treatment groups was assessed using two-way analysis of variance (ANOVA), with post hoc comparisons performed using Duncan’s Multiple Range Test (Duncan 1955). A significance level of P ≤ 0.05 was considered statistically significant.

## Results

### Isolation and identification of fungi from eggplant peel waste

**Fig. 1:**
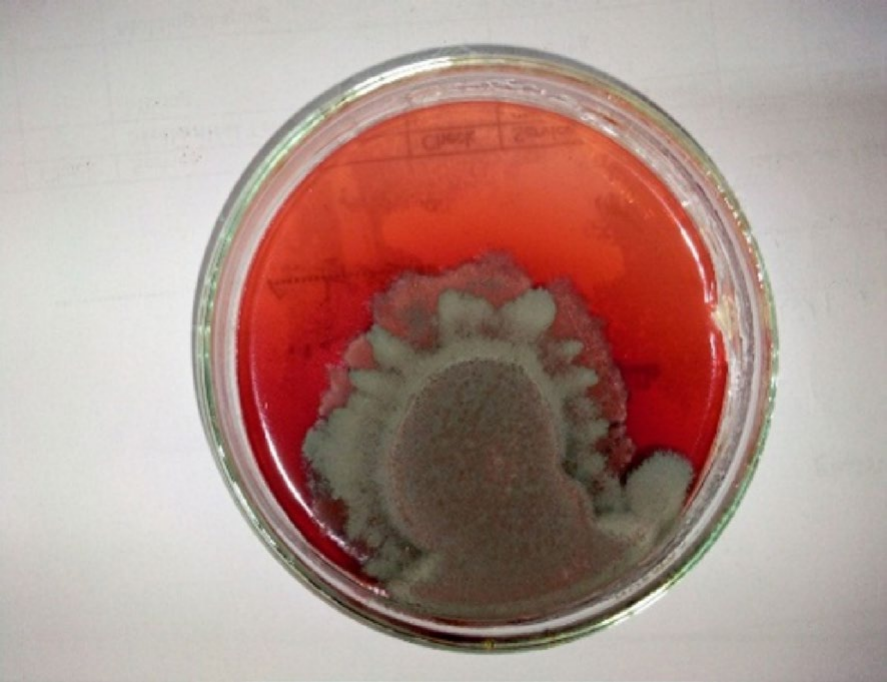
Morphology of *Talaromyces* sp. colony after 7 days on YES media.

The morphology of the fungal isolates producing pigment on YES media after culturing for 5 days at 28 ± 2 °C was shown in Fig. 1. Colonies are 12 mm in diameter, margins wide (2–3 mm), grayish-green color with white borders with a velvety texture, a dense sporulation. A diffusible red pigment was produced that was observed in the media.

**Fig. 2:**
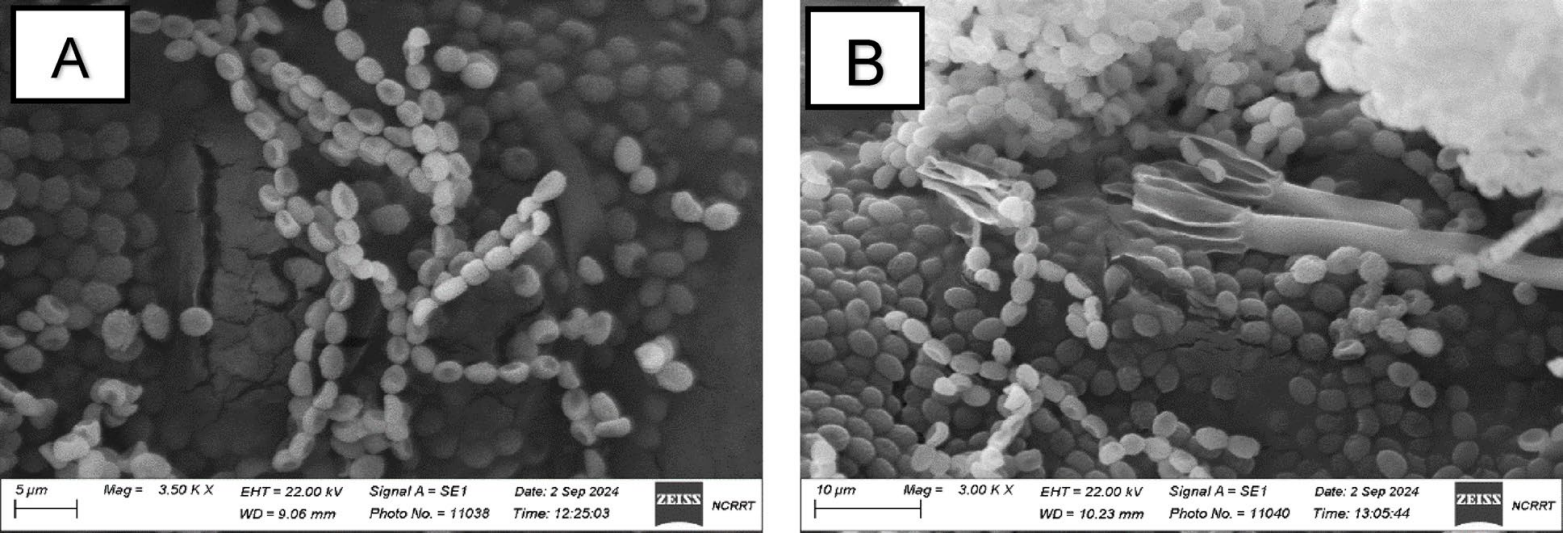
Morphology of *Talaromyces* sp colony under scanning electron microscope showing the thick-walled ellipsoidal conidia (A) and branching shape of conidiophores, biverticillate carrying ellipsoidal conidia (B)

**Fig. 3:**
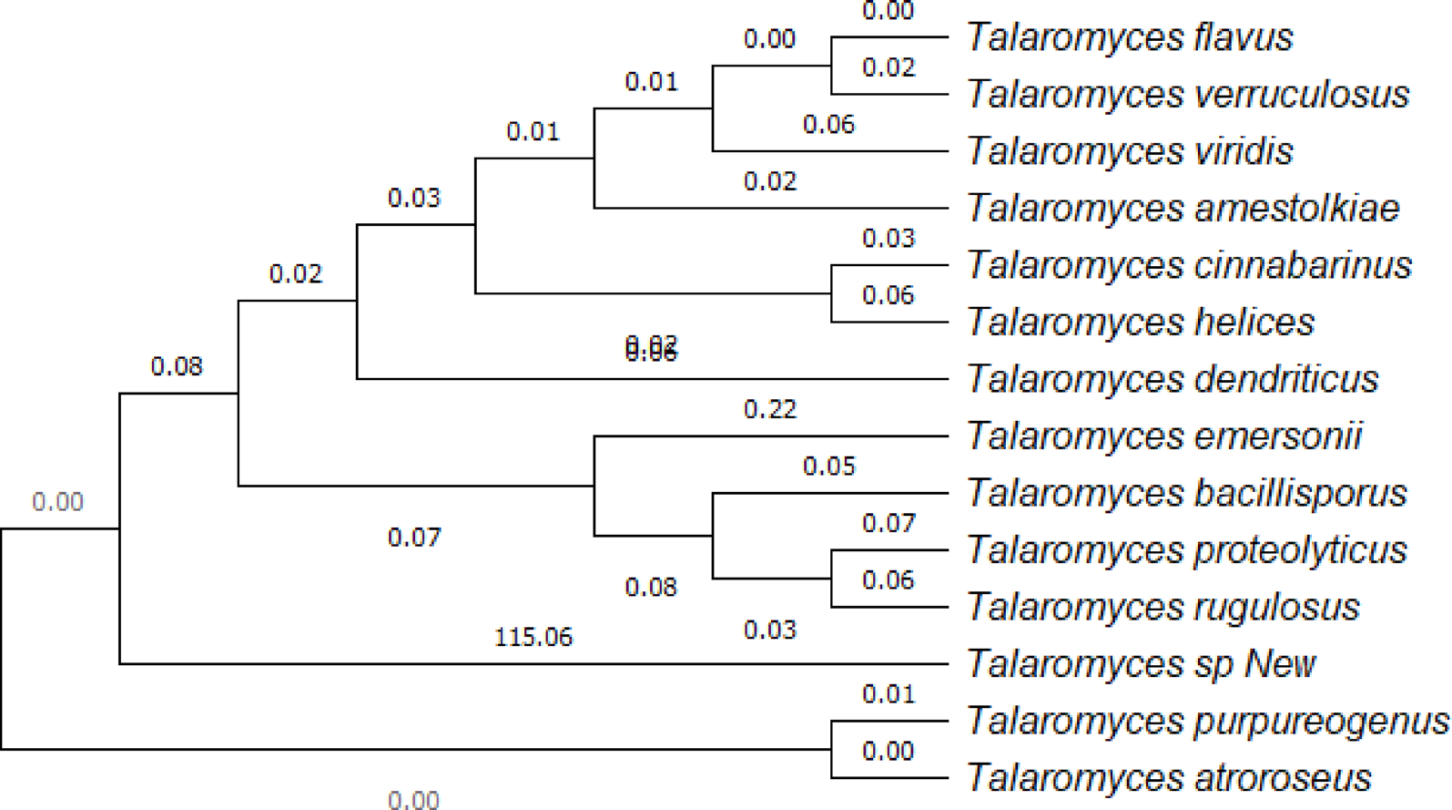
ITS phylogenetic identification of *Talaromyces* sp

Thick-walled ellipsoidal conidia with a rough appearance were reported by using the scanning electron microscope. There were typical branching conidiophore shapes, biverticillate (Fig. 2). The isolate was predicted to belong to *Talaromyces atroroseus*. The morphological identification was accompanied by molecular-based identification, to study the relatedness between the *Talaromyces atroroseus* species with other close species (Fig. 3).

After amplification of the ribosomal internal transcribed spacer rDNA of the fungal isolate using conventional PCR, *Talaromyces atroroseus* showed the closest similarity. The fungal sequence identified in this study was submitted to the NCBI GenBank database and was given accession number: PQ007745. The strain was also deposited as *Talaromyces atroroseus*, CCASU-2025-F13 in the Culture Collection Ain Shams University (CCASU).

In this study, based on the sequence of the ITS region, a *Talaromyces* phylogenetic analysis was performed. The constructed phylogenetic tree illustrated two major clusters. The first cluster included 11 ITS regions and belongs to the genus of *Talaromyces*, which showed a close relationship with each other, with different bootstrap values. The second cluster included the fungal isolate, which showed a close relationship with *Talaromyces atroroseus* and *Talaromyces purpureogenus* in the same clade, with a bootstrap value of 100%, as presented with others in the cluster (Fig. 3). The morphological characteristics of the fungal isolates (Figs. 1, 2) confirm their identification as *Talaromyces atroroseus*. This aligns with Salim et al. (2022), who reported identical morphological, phylogenetic, and pigment production traits for the strain under study.

### Electron beam irradiation on the mechanical tensile strength of eggplant peel waste

**Fig. 4:**
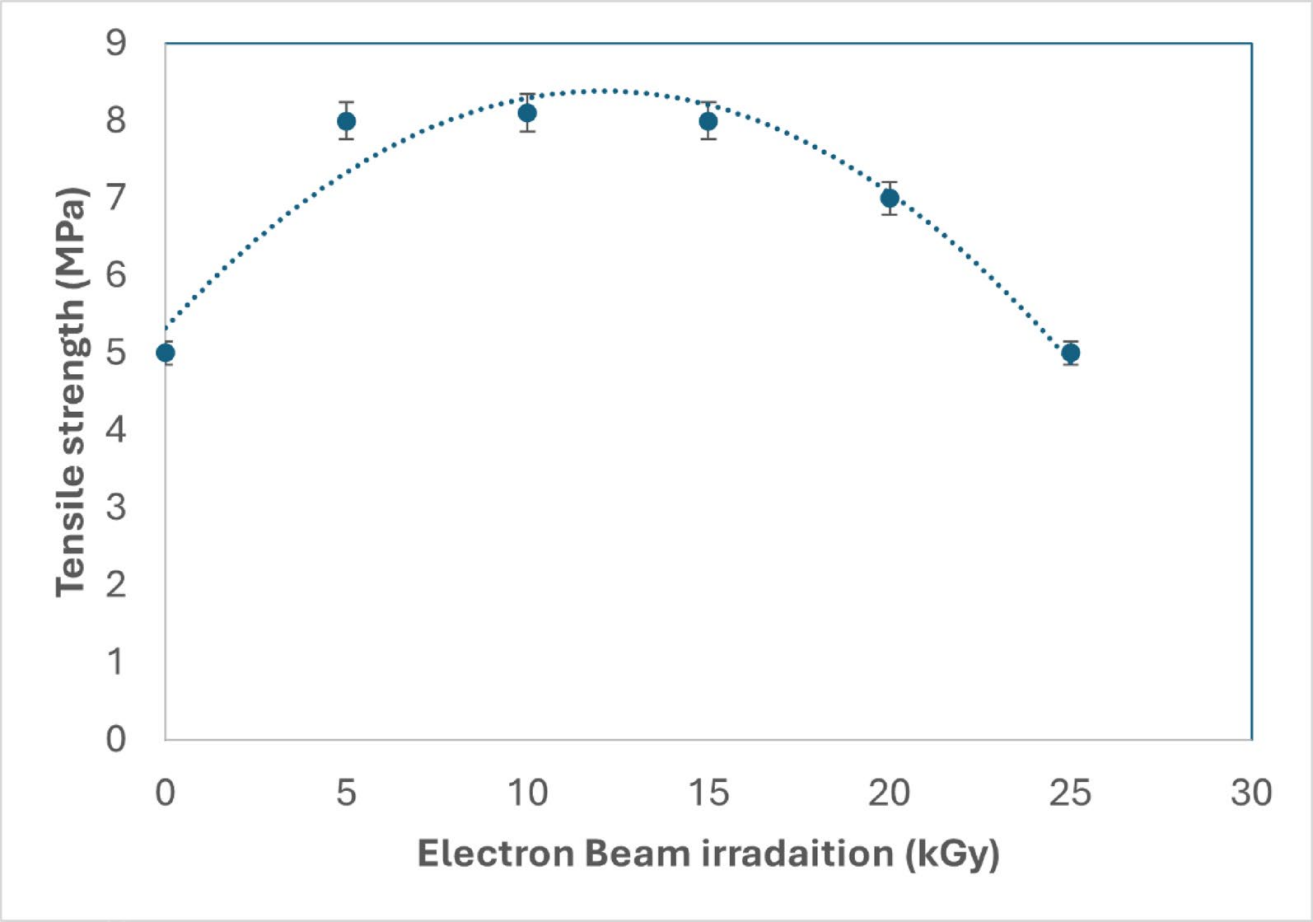
Tensile strength of electron beam irradiated eggplant peel waste

Electron beam irradiation significantly impacted mycoleather's tensile properties, as shown in Fig. 4. Tensile strength increased from 5 MPa (for unirradiated sample) to a peak of 8 MPa at 5, 10, and 15 kGy. Beyond this, it decreased to 7 MPa at 20 kGy and returned to 5 MPa at 25 kGy.

### Effect of electron beam irradiation on the microbial counts of eggplant peel waste

As shown in Table 1, electron beam irradiation effectively reduces microbial populations across all tested dose levels. Data analysis revealed that radiation processing at 5, 10, 15, 20, and 25 kGy led to around a 5-log reduction in total bacterial count and a 3-log reduction in total mold and yeast count.

**Table 1:** Total Microbial Counts of eggplant peels Samples treated with electron beam

	Effect of electron beam treatments on total aerobic mesophilic bacteria (Log CFU/g)					
	Control	5	10	15	20	25
Total aerobic mesophilic bacteria	5.6	ND	ND	ND	ND	ND

	Effect of electron beam treatments on total mold and yeast counts (Log CFU/g)					
	Control	5	10	15	20	25
Total mold and yeasts count	3.5	ND	ND	ND	ND	ND

ND, not detected

### Effect of electron beam irradiation on the sensory properties of eggplant peel waste

As shown in Table 2, increasing irradiation doses negatively impacted the sensory attributes of eggplant peels. Higher doses led to a decline in scores for color, texture, odor, and overall acceptability. Specifically, at 25 kGy, the peels developed a cooked odor, exhibited a dark brown color and mealy texture, and received the lowest overall acceptability ratings.

**Table 2:** Effect of electron beam irradiation treatments on the sensory evaluation

	Effect of electron beam irradiation treatments on the sensory scores of eggplant peels					
	Control	5	10	15	20	25
Color	4.0^a^_a_ ± 0.05	3.6^b^_a_ ± 0.01	3.4^c^_a_ ± 0.03	2.6^d^_a_ ± 0.05	2.0^e^_a_ ± 0.05	0.5^f^_a_ ± 0.03
Texture	4.0^a^_a_ ± 0.05	3.5^b^_a_ ± 0.03	3.2^c^_a_ ± 0.03	2.5^d^_a_ ± 0.05	1.9^e^_a_ ± 0.05	0.4^f^_a_ ± 0.03
Odor	4.0^a^_a_ ± 0.05	3.4^b^_a_ ± 0.05	3.1^c^_a_ ± 0.05	2.6^d^_a_ ± 0.03	1.9^e^_a_ ± 0.05	0.4^f^_a_ ± 0.08
Overall acceptability	4.0^a^_a_ ± 0.05	3.5^b^_a_ ± 0.05	3.2^c^_a_ ± 0.05	2.6^d^_a_ ± 0.03	2.0^e^_a_ ± 0.08	0.5^f^_a_ ± 0.12

Mean values followed by different superscripts (within rows) and different subscripts (within columns) are significantly different (*p* < 0.05)

### Optimization of exopolysaccharide production using full factorial design

**Fig. 5:**
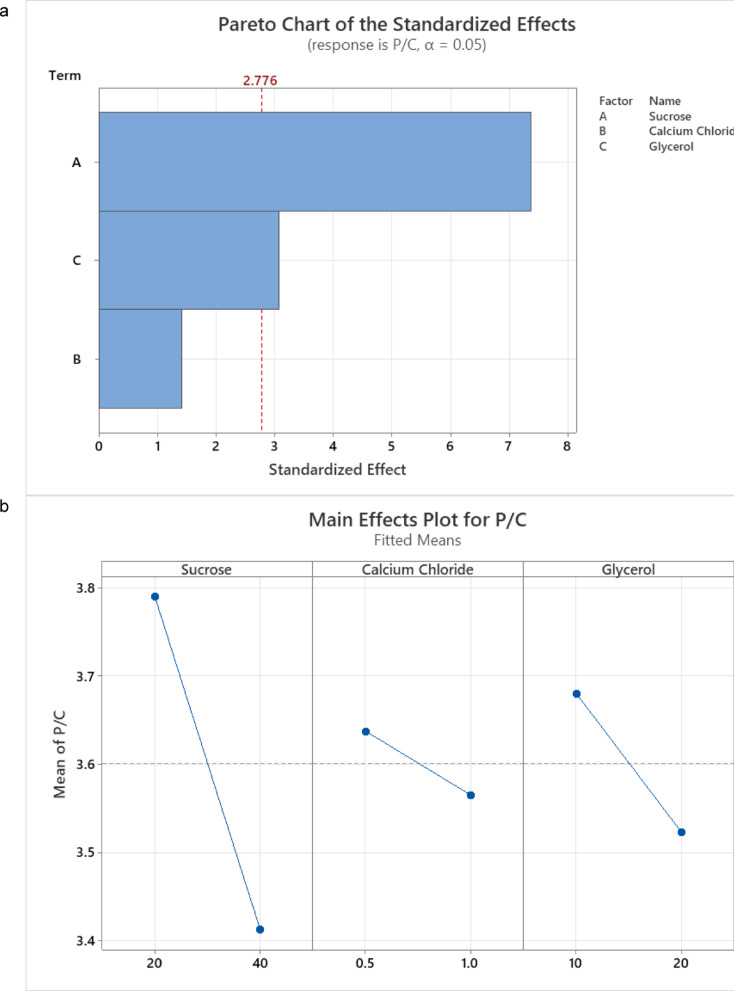
**a** Pareto chart for EPS under sucrose, calcium chloride and glycerol optimization. **b** Main effect plot for EPS under sucrose, calcium chloride and glycerol optimization conditions

In the present study, we evaluated three components—sucrose, calcium chloride, and glycerol—to the stickiness of the produced EPS, using the ranges and preparations detailed in S1. The resulting Pareto chart (Fig. 5a) indicates that sucrose is the primary contributor to EPS stickiness, followed by the addition of glycerol. Figure 5b further illustrates these main effects, showing a similar pattern to that obtained by the Pareto chart. The resulting R-squared value of 94.28% (S2) is considered a good fit for the model.

### Effect of different post-treatment methods on the spore destruction, morphological change, and thermal analysis of mycoleather

**Fig. 6:**
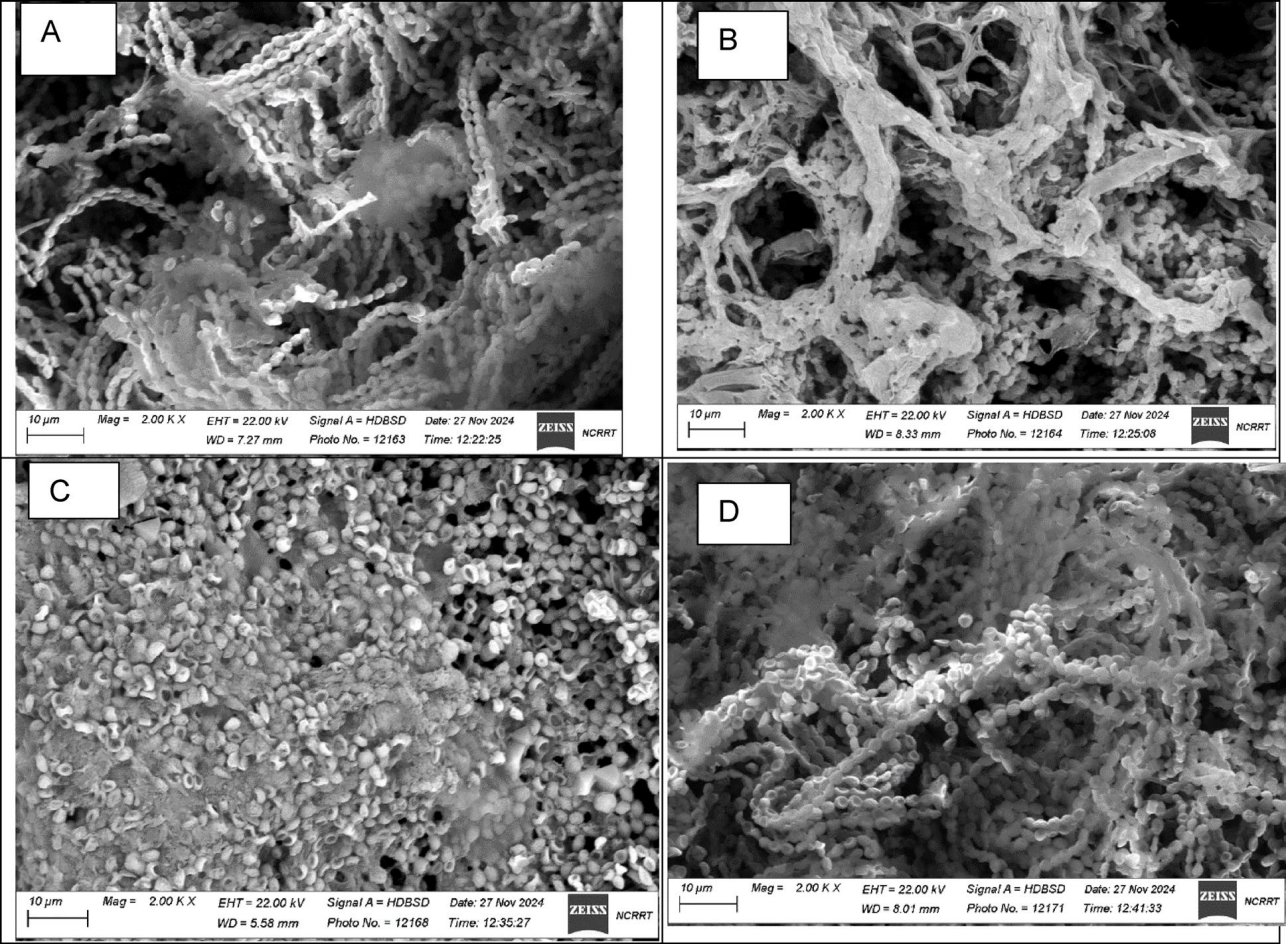
Scanning Electron Micrographs of Mycoleather after incubation at 40 °C (**A**), exposure to 5 kGy electron beam irradiation (**B**), exposure to UV (**C**) and immersion in sodium hypochlorite (**D**)

**Fig. 7:**
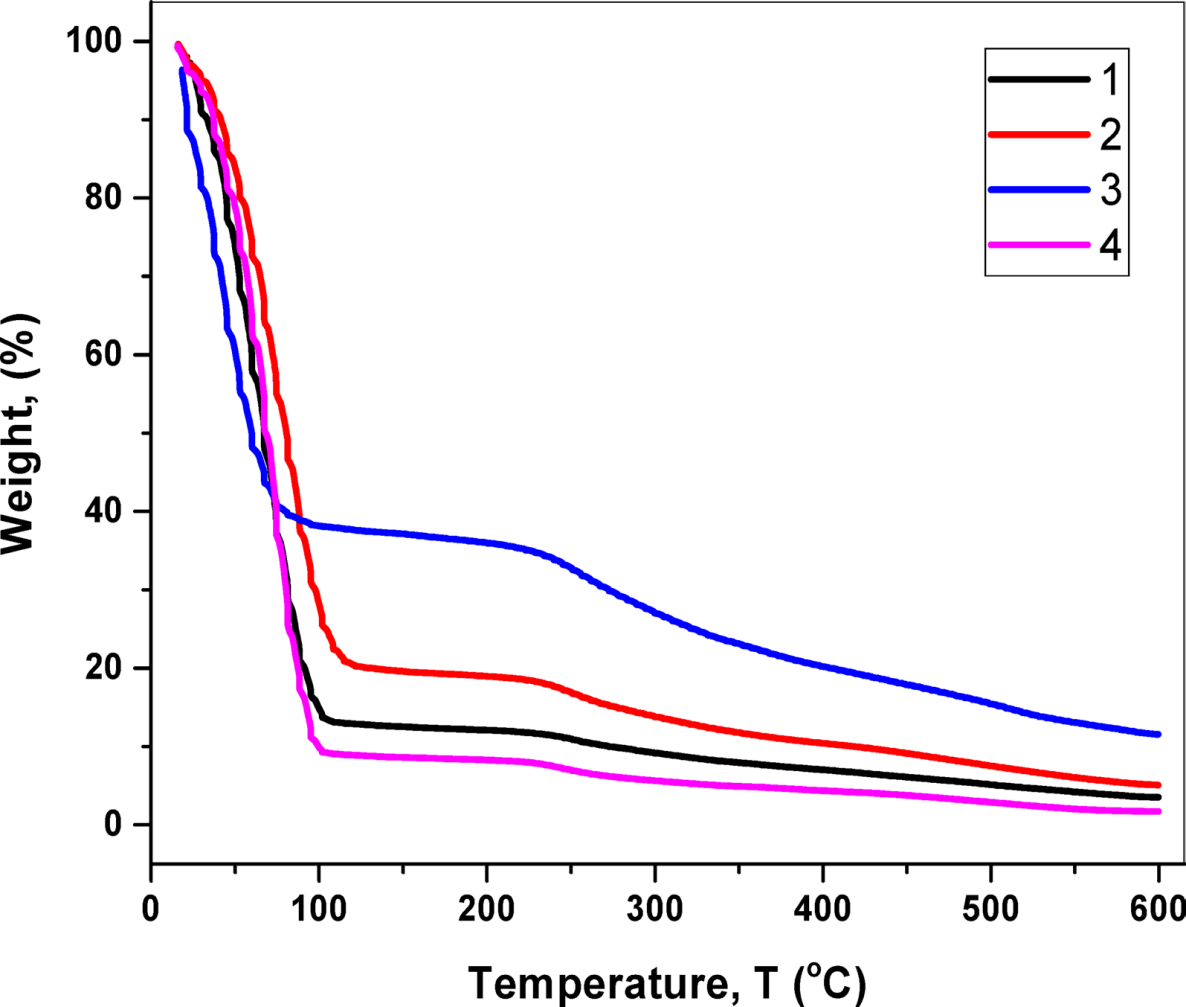
Thermal Gravimetric Analysis (TGA) of Mycoleather after incubation at 40 °C (1), exposure to 5 kGy electron beam irradiation (2), exposure to UV (3) and immersion in sodium hypochlorite (4)

Following full factorial design experiments and tensile strength analysis of irradiated eggplant peel, we prepared a new batch of mycelial leather for post-treatment evaluation. Various post-treatment approaches were explored to identify a suitable method for spore destruction without compromising the material's integrity. We tested post-incubation temperature, ionizing radiation, UV exposure, and immersion in sodium hypochlorite. All tested post-treatment methods successfully eliminated viable growth, as confirmed by the lack of microbial presence (Table 3). This indicates that each of these approaches can be effectively employed for decontamination. We also investigated the morphological changes in the mycoleather after each post-treatment. Incubation at 40 °C, exposure to ionizing radiation, and immersion in sodium hypochlorite did not alter the mycelial morphology of the produced material (Fig. 6a, b, d). However, UV post-treatment led to deformation of the mycelia, as depicted in Fig. 6c. Thermal gravimetric analysis (TGA) revealed similar thermal degradation patterns for samples treated with post-incubation temperature, ionizing radiation, and sodium hypochlorite. These samples exhibited an 80–90% weight reduction at 110 °C, followed by an additional 3% reduction at 240 °C. In contrast, UV post-treatment showed an initial 60% decrease at 95 °C, with a further 5% reduction at 250 °C (Fig. 7).

**Table 3:** Effect of post-treatment on viable count

	Effect of post-treatment on viable count (Log CFU/g)				
Control	Incubation at 40 °C		5 kGy	UV (254 nm)	5% Sodium hypochlorite
6.04	ND		ND	ND	ND

ND, not detected

## Discussion

The following work highlights the successful utilization of eggplant peel waste as a novel growth substrate for *Talaromyces atroroseus,* showing promising density under optimized conditions. This represents a novel approach in alternative leather production.

*Talaromyces atroroseus* was isolated from eggplant peel waste and was characterized as an exopolysaccharide-producing fungus that was promising for its use as a bio-binding material to produce mycoleather. *Talaromyces* *atroroseus* IBT 11181 was originally isolated from red sweet bell pepper bought at a supermarket as reported by Thrane et al. (2017). The morphological characteristics of the fungal isolates under study confirm the identification of fungal isolates to be *Talaromyces atroroseus*. This is due to Thick-walled ellipsoidal conidia on a typically branching shape of conidiophores, biverticillate, that differs from the main morphological characteristics of *Talaromyces purpureogenus* as shown by Yue et al. (2022), which shows spherical conidia at the top of the conidiophore’s broom-like branches. Many reports recommend *T. atroroseus* as an effective producer of the azaphilone biosynthetic families mitrorubrins and *Monascus-like* without any mycotoxins. In the literature, ITS sequencing as a fungal barcoding gene and PCR analysis were used as the most efficient strategy for the molecular identification of *T. verruculosus* (Khattab et al. 2020). Morphological characterization of the fungal isolate revealed its descriptive form in terms of the shape of the conidia, spores, and hyphae. Although morphological characteristics are useful for species description, they are limited by the infrequent macroscopic structures (Slepecky and Starmer 2009), other than cryptic species complexes that are present in many taxa (Hending 2025). Therefore, molecular tools were implemented to complement morphological ones. The tensile strength and observed elasticity of both the control and mycoleather samples might be the result of the mycelium filamentous networking in the eggplant peels. This finding was the same as Akhter et al. (2024). The strength of the mycoleather was highly competitive with cowhide leather, which showed tensile strength of 9.5 MPa (Kim et al. 2021). Mycelial-Based Leather showed variable tensile strength that varied from 7.21 to 8.49 MPa (Raman et al. 2022). Fungal textile with leather-like properties produced using *Rhizopus delemar* and bread waste showed tensile strengths of 6.9 MPa to 7.7 MPa depending on the binder and post-treatment (Wijayarathana et al. 2022). The effect of radiation in reducing the microbial count of minimally processed eggplant is attributed to direct or indirect effects of radiation on cells. In direct effect, inactivation of microorganisms by radiation is primarily due to DNA damage (Rahman et al. 2021). Direct radiation effect causes either single-strand or double-strand breaking of the DNA molecule, resulting in the inability of the cell to replicate, thus finally leading to death of the cell. Increased irradiation doses led to browning and an undesirable odor in the sensory evaluation of eggplant peels. These results are consistent with the observations of Hussain et al. (2014). Changes of color may be due to enzymatic browning on fresh cut vegetables and fruits (Moon et al. 2020), which may be correlated with polyphenol oxidase activity (Sui et al. 2023). The obtained results also showed an increase in red color of the mycelium mat of fungi with the eggplant peels, significantly with increasing radiation dose. This may be due to an increase in anthocyanin content of the eggplant peels. It is seen from the data that the increase in anthocyanin content was dose dependent. The increase in anthocyanin content following irradiation is thought to be either due to the production of initial burst of stimulatory amounts of ethylene, which in turn enhances the activities of phenyl alanine ammonia-lyase (PAL) and flavonoid glucosyl transferase (GT), the two key enzymes involved in the anthocyanin biosynthesis from phenyl-alanine (Sharma et al. 2022) or increase in the extractability of anthocyanins because of the changes in the cellular structure (EL-Samahyetal 2000; Moreno et al.^2007^). Thus, irradiation treatment of eggplant has proved beneficial in enhancing the release of biologically active constituents such as the pigments in eggplant peels. This result was the same as shown by Hussain et al. (2014). The three-dimensional interwoven mycelial network acts as a natural reinforcing material with the substrate (Medina et al. 2023), which in the current study is the eggplant peel. However, adding another bio-binder such as exopolysaccharides will result in enhancing this reinforcement. The media composition highly affects the exopolysaccharide production, which is directly related to the stickiness of the material. In the present study, three components were tested for their contribution to the stickiness of the produced EPS: sucrose, calcium chloride, and glycerol. The obtained results show that sucrose contributes highly to the stickiness of EPS; this was followed by the addition of glycerol to the media. The increase in sugar concentration can be directly related to both surface-bound and released exopolysaccharides in the media (Gomaa et al. 2022). On the other hand, glycerol has two roles. The first is its contribution as sugar alcohol in the media, and the second is its role as plasticizer. It has been reported to enhance elastic properties of fungal leather (Wijayarathna et al. 2022). Glycerol was reported to increase plasticity, an important feature needed in leather manufacturing. A low tensile strength is preferred for plasticized material since it gives it more flexibility (Inyati et al. 2019). Adding calcium chloride to the culture media did not result in increasing the stickiness as opposed to what was initially hypothesized. Generally, adding divalent cations increases cross-linking by binding to the free carboxyl group of some amino acids, which results in salt bridges. They have been reported to contribute to surface hydrophobicity (Ai et al. 2020). The addition of calcium chloride was reported to increase the excretion of EPS into the media since it increases cell membrane permeability (Mahapatra and Benerjee 2013; Gomaa et al. 2013). On the other hand, Zhang et al. (2022) reported that increasing the concentration of Ca^2+^ can result in a decrease in the overall viscosity and thus the concentration plays a significant role in hydrophobic interactions, disulfide bonding, and hydrogen bonding. Optimizing EPS production is important for microbial metabolite production, especially if it will be used in an industrial application. Using biotechnological technologies to generate fiber-based materials strengthens the potential for innovation chances. Fungal mycelium fiber networks consist of cellulose, chitin, and proteoglycans and can grow on organic waste (Papp et al. 2017; Meyer 2021). Enhancing EPS and stickiness ensures binding of the material and its mechanical properties (Ibrahim et al. 2024). Based on the full factorial design experiment and the tensile strength of irradiated eggplant peel, a new batch of mycoleather was prepared and was exposed to post-treatment. The different post-treatment approaches were tested to depict a suitable method to ensure destruction of spores without affecting the material itself. Post-incubation temperature, ionizing radiation, UV, and sodium hypochlorite were all tested. The results show that all approaches resulted in a lack of viable growth, which ensures that all post-treatment approaches can be used. The results showed that incubating the material at 40 °C, exposure to ionizing radiation, and immersion in sodium hypochlorite did not alter mycelial morphology for the produced material. On the other hand, post-treatment using UV resulted in deformation of the mycelia. TGA is a widely used thermal technique that is used to study the change in material mass as a function of temperature and is considered important for studying polymeric materials and their decomposition patterns. The initial drop in mass is due to the loss of plasticizer in the polymer, while the following mass loss takes place due to loss of residual polymer. The pattern obtained in the present work is similar to that obtained by Wijayarathana et al. (2022) where they stated that initial mass reduction which takes place between 25 and 200 °C was attributed to evaporation of moisture and chemically bonded water molecules, while the mass reduction in the second region from 200 to 375 °C correlates to the decomposition of organic compounds such as chitin, proteins and polysaccharides. However, our results did not show a third mass loss region, which is usually from 375 to 600 °C. Mycoleather, which is thermally stable at > 200 °C is considered of acceptable quality (Bustillos et al. 2020).

## Conclusion

Biotechnology offers crucial applications for waste valorization. This study successfully harnessed fungi grown on food waste to produce an alternative leather. We demonstrated that ionizing radiation could effectively modify the tactile and mechanical properties of eggplant peel waste. Our findings show that *Talaromyces* sp. grown under optimized conditions could yield a mycoleather with good density. Furthermore, we determined that a post-treatment dose of 5 kGy of ionizing radiation could effectively produce spore-free mats. The growing interest in sustainable, alternative leather products, already adopted by major sportswear and haute couture brands, highlights the significance of this work. This novel approach, particularly the incorporation of eggplant peel waste—a first to the authors' knowledge—is poised to contribute significantly to a sustainable and circular economy, ultimately aiding in the reduction of greenhouse gases. The present study demonstrates proof of concept that paves the way for mycoleather commercialization. However, further research is required to leverage mycoleather production by integrating Artificial Intelligence (AI), omics, and nanotechnology. Additional evaluation is essential to assess Life cycle Assessment (LCA) and Techno-Economic analysis, long-term durability, and biological safety. Overall, this work marks a significant step towards ecofriendly and sustainable production.

## Supplementary Information

Below is the link to the electronic supplementary material.

Supplementary Material 1.

Supplementary Material 2.

## Data Availability

No datasets were generated or analysed during the current study.

## Abbreviations

CAGR: Compound annual growth rate
CCASU: Culture Collection Ain Shams University
CYA: Czapek’s yeast extract agar medium
EPS: Exopolysaccharides
FAO: Food and Agriculture Organization
GHGs: Greenhouse gases
ICT: Insulated Core Tank
NCRRT: National Center for Radiation Research and Technology
SDA: Sabouraud dextrose agar
SEM: Scanning electron microscope
DOE: Design of experiment
TGA: Thermal gravimetric analysis
TBC: Total bacterial count
TMYC: Total mold and yeast count
YES: Yeast extract sucrose

## References

[CR1] Abd El-kader R, Abdel-Khalek HH, Hammad A, Youssef KH, Abdou D (2020) Growth inhibition of aflatoxigenic molds and biodegradation of aflatoxin B1 by certain bacterial isolates. Egypt J Microbiol 55:63–77. 10.21608/ejm.2020.37114.1166

[CR2] Agregán R, Munekata PES, Feng X, Astray G, Gullón B, Lorenzo JM (2021) Recent advances in the extraction of polyphenols from eggplant and their application in foods. LWT 146:11381. 10.1016/J.LWT.2021.111381

[CR3] Ai M, Zhou Q, Guo S, Fan H, Cao Y, Ling Z, Zhou L, Jiang A (2020) Characteristics of intermolecular forces, physicochemical, textural and microstructural properties of preserved egg white with Ca(OH)_2_ addition. Food Chem 314:126206. 10.1016/j.foodchem.2020.126206 31951888 10.1016/j.foodchem.2020.126206

[CR4] Akhter S, Jahan MS, Rahman ML, Ruhane TA, Ahmed M, Khan MA (2024) Revolutionizing sustainable fashion: jute-mycelium vegan leather reinforced with polyhydroxyalkanoate biopolymer crosslinking from novel bacteria. Adv Polym Tech. 10.1155/2024/1304800

[CR5] Amobonye A, Lalung J, Awasthi MK, Pillai S (2023) Fungal mycelium as leather alternative: a sustainable biogenic material for the fashion industry. Sustain Mater Technol. 10.1016/j.susmat.2023.e00724

[CR6] APHA (1992) Standard methods for the examination of water and wastewater, 18th edn. American Public Health Association (APHA), American Water Works Association (AWWA) and Water Pollution Control Federation (WPCF), Washington DC

[CR8] Bustillos J, loganathan A, Agrawal R, Gonzalez B, Ramaswamy S, Boesl B, Agarwal A (2020) Uncovering the mechanical, thermal, and chemical characteristics of biodegradable mushroom leather with intrinsic antifungal and antibacterial properties. ACS Appl Bio Mater 3(5):3145–3156. 10.1021/acsabm.0c00164 35025358 10.1021/acsabm.0c00164

[CR9] Crawford A, Miller SR, Branco S, Fletcher J, Stefanov D (2024) Growing mycelium leather: a paste substrate approach with post-treatments. Res Dir Biotechnol Design 2:e6. 10.1017/btd.2024.6

[CR10] Duncan DB (1955) Multiple range and multiple F-test. Biometrics 11:1–5

[CR11] ELSamahy SK, Youssef BM, Askar AA, Swailam HMM (2000) Microbiological and chemical properties of irradiated mango. J Food Saf 20:39–156. 10.1111/j.1745-4565.2000.tb00294.x

[CR12] Enriquez-Medina I, Bermudez AC, Ortiz-Montoya EY, Alvarez-Vasco C (2023) From purposeless residues to biocomposites: A hyphae made connection. Biotechnol Rep (Amst) 1(39):e00807. 10.1016/j.btre.2023.e00807 10.1016/j.btre.2023.e00807 PMC10338154 37448784

[CR13] FAO (2019) The state of food and agriculture—moving forward on food loss and waste reduction. 10.4324/9781315764788

[CR14] FAOSTAT (2023) Accessed from 13 Jan 2023, from https://fao.org/faostat/en/#data/QCL

[CR15] Gomaa OM, Selim NS, Linz JE (2013) A possible role of *Aspergillus niger* mitochondrial cytochrome c in malachite green reduction under calcium chloride stress. Cell Biochem Biophys 67:1291–1299. 10.1007/s12013-013-9661-1 23737340 10.1007/s12013-013-9661-1

[CR16] Gomaa OM, Jassim AY, Chanda A (2022) Bioremoval of PVP-coated silver nanoparticles using *Aspergillus niger*: the role of exopolysaccharides. Environ Sci Pollut Res Int 29(21):31501–31510. 10.1007/s11356-021-18018-9 35001269 10.1007/s11356-021-18018-9 PMC8743098

[CR17] Grand view research. https://www.grandviewresearch.com/industry-analysis/leather-goods-market. Leather Goods Market Size, Share & Trends Analysis Report By Type (Genuine Leather, Synthetic Leather, Vegan Leather), By Product, By Region, And Segment Forecasts, 2023– 2030. Report ID: GVR-3-68038-061-3

[CR18] Gürbüz N, Uluisik S, Frary A, Frary A, Do˘ganlar S, (2018) Health benefits and bioactive compounds of eggplant. Food Chem 268:602–610. 10.1016/j.foodchem.2018.06.093 30064803 10.1016/j.foodchem.2018.06.093

[CR19] Hending D (2025) Cryptic species conservation: a review. Biol Rev 100(1):258–274. 10.1111/brv.13139 39234845 10.1111/brv.13139 PMC11718601

[CR20] Horincar G, Enachi E, Barbu V, Andronoiu DG, Râpeanu G, Stănciuc N, Aprodu I (2020) Value-added pastry cream enriched with microencapsulated bioactive compounds from eggplant (*Solanum melongena* L.) peel. Antioxidants (Basel, Switzerland) 9(4):351. 10.3390/antiox9040351 32340388 10.3390/antiox9040351 PMC7222404

[CR21] Hussain Peerzada R, Omeera A, Suradkar Prashant P, Dar Mohd A (2014) Effect of combination treatment of gamma irradiation and ascorbic acid on physicochemical and microbial quality of minimally processed eggplant (*Solanum melongena* L.). Radiat Phys Chem 103:131–141. 10.1016/j.radphyschem.2014.05.063

[CR22] Ibrahim RA, Fahim IS, Shaban M, Gomaa OM (2024) sustainable wood composite production using cotton waste and exopolysaccharides as green binder. Int J Biol Macromol 281:135710. 10.1016/j.ijbiomac.2024.135710 39419676 10.1016/j.ijbiomac.2024.135710

[CR23] Ikram M (2022) Transition toward green economy: technological innovation’s role in the fashion industry. Curr Opin Green Sustain Chem. 10.1016/j.cogsc.2022.100657

[CR24] Inayati I, Pamungkas DJ, Matovanni MPN (2019) Effect of glycerol concentration on mechanical characteristics of biodegradable plastic from rice straw cellulose. AIP Conf Proc 2097(1):030110. 10.1063/1.5098285

[CR25] Kazemi M, Khodaiyan F, Hosseini SS (2019a) Eggplant peel as a high potential source of high methylated pectin: ultrasonic extraction optimization and characterization. LWT 105:182–189. 10.1016/j.lwt.2019.01.060

[CR26] Kazemi M, Khodaiyan F, Hosseini SS (2019b) Utilization of food processing wastes of eggplant as a high potential pectin source and characterization of extracted pectin. Food Chem 294:339–346. 10.1016/j.foodchem.2019.05.063 31126472 10.1016/j.foodchem.2019.05.063

[CR28] Khattab OK, Ismail SA, Abosereh NA, Abo-Elnasr AA, Nour SA, Hashem AM (2020) Optimization and comparative studies on activities of β-mannanase from newly isolated fungal and its mutant. Egypt Pharm J 19(1):29–46. 10.4103/epj.epj_48_19

[CR29] Kim H, Song JE, Kim HR (2021) Comparative study on the physical entrapment of soy and mushroom proteins on the durability of bacterial cellulose bio-leather. Cellulose 28(5):3183–3200. 10.1007/s10570-021-03705-0

[CR30] Lazăr N, Râpeanu G, Iticescu C (2024) Mitigating eggplant processing waste’s environmental impact through functional food developing. Trends Food Sci Technol 147:104414. 10.1016/j.tifs.2024.104414

[CR31] Liu X, Zhang X, Wang X, Yue O, Jiang H (2024) Engineered, environmentally friendly leather-like bio-based materials. Trends Biotechnol. 10.1016/j.tibtech.2024.11.006 39616086 10.1016/j.tibtech.2024.11.006

[CR32] Maghrawy HH, Abd El Kareem H, Gomaa OM (2024) Enhanced exopolysaccharide production in gamma irradiated *Bacillus subtilis*: a biofilm-mediated strategy for ZnO nanoparticles removal. Int J Biol Macromol 258:128884. 10.1016/j.ijbiomac.2023.128884 38141708 10.1016/j.ijbiomac.2023.128884

[CR33] Mahapatra S, Banerjee D (2013) Fungal exopolysaccharide: production, composition and applications. Microbiol Insights 6:MBI-S10957 10.4137/MBI.S10957 PMC3987751 24826070

[CR34] Majeed H, Iftikhar T (2024) Ecofriendly reactive printing of cellulosic fabric with sustainable novel techniques. Cellulose 31(11):7067–7081. 10.1007/s10570-024-06008-2

[CR35] Majeed H, Iftikhar T, Ahmad K, Qureshi K, Altaf F, Iqbal A, Khalid A (2023) Bulk industrial production of sustainable cellulosic printing fabric using agricultural waste to reduce the impact of climate change. Int J Biol Macromol 253:126885. 10.1016/j.ijbiomac.2023.126885 37709213 10.1016/j.ijbiomac.2023.126885

[CR36] Mauro RP, Agnello M, Rizzo V, Graziani G, Fogliano V, Leonardi C, Giuffrida F (2020) Recovery of eggplant field waste as a source of phytochemicals. Sci Hortic 261:109023. 10.1016/j.scienta.2019.109023

[CR37] Meyer M, Dietrich S, Schulz H, Mondschein A (2021) Comparison of the technical performance of leather, artificial leather, and trendy alternatives. Coatings 11(2):226. 10.3390/coatings11020226

[CR38] Moon KM, Kwon EB, Lee B, Kim CY (2020) Recent trends in controlling the enzymatic browning of fruit and vegetable products. Molecules 25(12):2754. 10.3390/molecules25122754 32549214 10.3390/molecules25122754 PMC7355983

[CR39] Moreno M, Castell-Perez ME, Gomes CL, Silva PFD, Kim J, Moreira RG (2007) Optimizing electron beam irradiation of “tommy atkins” mangoes (*Mangifera indica* L.). J Food Process Eng 30(4):436–457. 10.1111/j.1745-4530.2007.00111.x

[CR40] Nayak A, Bhushan B (2019) An overview of the recent trends on the waste valorization techniques for food wastes. JEM 233:352–370. 10.1016/j.jenvman.2018.12.041 10.1016/j.jenvman.2018.12.041 30590265

[CR41] Papamichael I, Chatziparaskeva G, Pedreño JN, Voukkali I, Almendro Candel MB, Zorpas AA (2022) Building a new mind set in tomorrow fashion development through circular strategy models in the framework of waste management. Curr Opin Green Sustain Chem 36:100638. 10.1016/j.cogsc.2022.100638

[CR42] Papp N, Rudolf K, Bencsik T, Czégényi D (2017) Ethnomycological use of *Fomes fomentarius* (L.) Fr. and *Piptoporus betulinus Bull*. P. Karst in transylvania, Romania. Genet Resour Crop Evol 64(1):101–111. 10.1007/s10722-015-0335-2

[CR43] Rahman M, Islam MA, Das KC, Salimullah M, Mollah MZI, Khan RA (2021) Effect of gamma radiation on microbial load, physico-chemical and sensory characteristics of common spices for storage. J Food Sci Technol 58(9):3579–3588. 10.1007/s13197-021-05087-4 34366475 10.1007/s13197-021-05087-4 PMC8292502

[CR44] Raman J, Kim DS, Kim HS, Oh DS, Shin HJ (2022) Mycofabrication of mycelium-based leather from brown-rot fungi. J Fungi 8(3):317. 10.3390/jof8030317 10.3390/jof8030317 PMC8950489 35330319

[CR45] Roh EK (2020) Mechanical properties and preferences of natural and artificial leathers, and their classification with a focus on leather for bags. J Eng Fibers Fabr. 10.1177/1558925020968825

[CR56] Sajn N (2019) Environmental impact of the textile and clothing industry: what consumers need to know. European Parliamentary Research Service. 633143. available at: https://www.europarl.europa.eu/thinktank/en/document/EPRS_BRI

[CR46] Salim RG, Fadel M, Youssef YA, Taie HA, Abosereh NA, El-Sayed GM, Marzouk M (2022) A local *Talaromyces atroroseus* TRP-NRC isolate: isolation, genetic improvement, and biotechnological approach combined with LC/HRESI-MS characterization, skin safety, and wool fabric dyeing ability of the produced red pigment mixture. J Genet Eng Biotechnol 20(1):62. 10.1186/s43141-022-00335-2 35451646 10.1186/s43141-022-00335-2 PMC9033925

[CR47] Sharma H, Chawla N, Dhatt AS (2022) Role of phenylalanine/tyrosine ammonia lyase and anthocyanidin synthase enzymes for anthocyanin biosynthesis in developing *Solanum melongena* L. genotypes. Physiol Plant 174(5):e13756. 10.1111/ppl.13756 36281844 10.1111/ppl.13756

[CR48] Shirvanimoghaddam K, Motamed B, Ramakrishna S, Naebee M (2020) Death by waste: fashion and textile circular economy case. Sci Total Environ. 10.1016/j.scitotenv.2020.137317 32088483 10.1016/j.scitotenv.2020.137317

[CR49] Slepecky RA, Starmer WT (2009) Phenotypic plasticity in fungi: a review with observations on *Aureobasidium pullulans*. Mycologia 101:823–832. 10.3852/08-197 19927747 10.3852/08-197

[CR55] Sui X, Meng Z, Dong T, Fan X, Wang Q (2023) Enzymatic browning and polyphenol oxidase control strategies. Curr Opin Biotechnol 81:102921. 10.1016/j.copbio.2023.102921 10.1016/j.copbio.2023.102921 36965297

[CR50] Thrane U, Rasmussen KB, Petersen B, Rasmussen S, Sicheritz-Pontén T, Mortensen UH (2017) Genome sequence of *Talaromyces atroroseus*, which produces red colorants for the food industry. Genome Announc 5(9):10. 10.1128/genomeA.01736-16 10.1128/genomeA.01736-16 PMC5334594 28254987

[CR51] Wijayarathna EKB, Mohammadkhani G, Soufiani AM, Adolfsson KH, Ferreira JA, Hakkarainen M, Berglund L, Heinmaa I, Root A, Zamani A (2022) Fungal textile alternatives from bread waste with leather-like properties. Resour Conserv Recycl 179:106041. 10.1016/j.resconrec.2021.106041

[CR52] Woodside AG, Fine MB (2019) Sustainables fashion themes in luxury brand storytelling: the sustainability fashion research grid. J Glob Fash Mark 10:111–128. 10.1080/20932685.2019.1573699

[CR53] Yue Y, Jiang M, Hu H, Wu J, Sun H, Jin H, Hou T, Tao K (2022) Isolation, identification and insecticidal activity of the secondary metabolites of *Talaromyces purpureogenus* BS5. J Fungi (Basel) 8(3):288. 10.3390/jof8030288 35330290 10.3390/jof8030288 PMC8949156

[CR54] Zhang J, Jiang L, Yang J, Chen X, Shen M, Yu Q, Chen Y, Xie J (2022) Effect of calcium chloride on heat-induced *Mesona chinensis* polysaccharide-whey protein isolation gels: gel properties and interactions. LWT 155:112907. 10.1016/j.lwt.2021.112907

