# Supplementary material for: Mycofabrication of sustainable mycelium-based leather using Talaromyces sp. and irradiated eggplant peel waste
Source: AMB Express. 2025 Aug 22;15:124. doi: 10.1186/s13568-025-01935-0 (PMC12373593; doi:10.1186/s13568-025-01935-0)
Supplement: Supplementary file 1 — Supplementary Material 1. [file 13568_2025_1935_MOESM1_ESM.docx]

**Design Table**

| **Run** | **Blk** | **Sucrose (g/L)** | **Calcium Chloride (M)** | **Glycerol (%)** |
| --- | --- | --- | --- | --- |
| 1 | 1 | 40 | 1 | 10 |
| 2 | 1 | 20 | 1 | 20 |
| 3 | 1 | 40 | 1 | 20 |
| 4 | 1 | 20 | 1 | 10 |
| 5 | 1 | 20 | 0.5 | 10 |
| 6 | 1 | 20 | 0.5 | 20 |
| 7 | 1 | 40 | 0.5 | 10 |
| 8 | 1 | 40 | 0.5 | 20 |
