# Supplementary material for: Mycofabrication of sustainable mycelium-based leather using Talaromyces sp. and irradiated eggplant peel waste
Source: AMB Express. 2025 Aug 22;15:124. doi: 10.1186/s13568-025-01935-0 (PMC12373593; doi:10.1186/s13568-025-01935-0)
Supplement: Supplementary file 2 — Supplementary Material 2. [file 13568_2025_1935_MOESM2_ESM.docx]

**Coded Coefficients**

| **Term** | **Effect** | **Coef** | **SE Coef** | **T-Value** | **P-Value** | **VIF** |
| --- | --- | --- | --- | --- | --- | --- |
| Constant |  | 3.6012 | 0.0256 | 140.75 | 0.000 |  |
| Sucrose | -0.3775 | -0.1888 | 0.0256 | -7.38 | 0.002 | 1.00 |
| Calcium Chloride | -0.0725 | -0.0363 | 0.0256 | -1.42 | 0.230 | 1.00 |
| Glycerol | -0.1575 | -0.0787 | 0.0256 | -3.08 | 0.037 | 1.00 |

**Model Summary**

| **S** | **R-sq** | **R-sq(adj)** | **R-sq(pred)** |
| --- | --- | --- | --- |
| 0.0723706 | 94.28% | 89.99% | 77.11% |

**Analysis of Variance**

| **Source** | **DF** | **Adj SS** | **Adj MS** | **F-Value** | **P-Value** |
| --- | --- | --- | --- | --- | --- |
| Model | 3 | 0.34514 | 0.115046 | 21.97 | 0.006 |
| Linear | 3 | 0.34514 | 0.115046 | 21.97 | 0.006 |
| Sucrose | 1 | 0.28501 | 0.285012 | 54.42 | 0.002 |
| Calcium Chloride | 1 | 0.01051 | 0.010512 | 2.01 | 0.230 |
| Glycerol | 1 | 0.04961 | 0.049612 | 9.47 | 0.037 |
| Error | 4 | 0.02095 | 0.005237 |  |  |
| Total | 7 | 0.36609 |  |  |  |

**Regression Equation in Uncoded Units**

| P/C | = | 4.513 - 0.01888 Sucrose - 0.145 Calcium Chloride - 0.01575 Glycerol |
| --- | --- | --- |
